# Supplementary material for: Social transmission in the wild can reduce predation pressure on novel prey signals
Source: Nat Commun. 2021 Jun 25;12:3978. doi: 10.1038/s41467-021-24154-0 (PMC8233390; doi:10.1038/s41467-021-24154-0)
Supplement: Supplementary file 1 — Supplementary Information [file 41467_2021_24154_MOESM1_ESM.pdf]

Supplementary Information for

Social transmission in the wild can reduce predation pressure on novel prey signals

Liisa Hämäläinen[1,2,3*], William Hoppitt[4], Hannah M. Rowland[1,5,6], Johanna Mappes[3], Anthony J. Fulford[1], Sebastian Sosa[7] & Rose Thorogood[1,8,9]

[1]Department of Zoology, University of Cambridge, UK

[2]Department of Biological Sciences, Macquarie University, NSW, Australia

[3]Department of Biological and Environmental Sciences, University of Jyväskylä, Finland

[4]School of Biological Sciences, Royal Holloway, University of London, Egham, UK

[5]Max Planck Institute for Chemical Ecology, Jena, Germany

[6]Institute of Zoology, Zoological Society of London, UK

[7]University of Strasbourg, CNRS, IPHC UMR 7178, Strasbourg, France

[8]HiLIFE Helsinki Institute of Life Sciences, University of Helsinki, Finland

[9]Research Programme in Organismal & Evolutionary Biology, Faculty of Biological and Environmental Sciences, University of Helsinki, Finland

*Correspondence:
liisa.hamalainen@mq.edu.au

Contents

1. Supplementary Methods and Results
2. Supplementary Figures
3. Supplementary Tables
4. Supplementary References

1. Supplementary Methods and Results

Discriminability of colours

We conducted the learning experiment with three different colour pairs. To investigate discriminability of colours in each pair, we took photographs of the coloured almonds (using Canon Ixus PC2052 Digital Camera) and measured their RGB values using Adobe Photoshop. We then calculated colour contrast ratios based on these values. These contrast ratios were similar in each colour pair: 1.97 in red/green (RGB: red = 211,57,33; green = 115,184,57), 1.77 in blue/purple (RGB: blue = 3,171,200; purple = 187,77,93) and 1.82 in yellow/orange (RGB: yellow = 226,240,99; orange = 228,158,67). This suggests that discriminability of colours was similar in all learning experiments.

First foraging choices in avoidance learning experiments

When investigating the first choice of each bird that visited the feeders on the first day of the experiment, we found that birds had a slight preference for green almonds in the red/green experiment (25 birds visited green and 13 red as their first choice; binomial test 25/38, p = 0.07), but no initial preferences in blue/purple (25 birds visited purple and 36 blue as their first choice; binomial test 25/61, p = 0.20) or yellow/orange experiments (63 birds visited orange and 69 yellow as their first choice; binomial test 63/132, p = 0.66).

Social information use during avoidance learning: simulations to validate the model

Because we could not measure the real number of observed feeding events, we reasoned that the probability that one individual i, observes a specific feeding event by another individual j, is proportional to the network connection between them, a_{ij}. We then calculated the expected number of negative feeding events observed, prior to each choice (occurring at time t) as

$$O_{-,i}(t) = \sum_j N_{-,j}(t) a_{ij,} \qquad (1)$$

where $N_{-,j}(t)$ was the number of times *j* had visited unpalatable almonds prior to time *t*, and summation is across all birds in the network, and likewise for the expected number of positive feeding events:

$$O_{+,i}(t) = \sum_j N_{+,j}(t) a_{ij}, \qquad (2)$$

where $N_{+,j}(t)$ was the number of times *j* had visited palatable almonds prior to time *t*. We then modelled the probability of *i* choosing the unpalatable option at time *t* as:

$$p_{-,i}(t) = \text{logit}\big(\alpha + \beta_{\text{asoc}+} N_{+,i}(t) + \beta_{\text{asoc}-} N_{-,i}(t) + \beta_{\text{soc}+} O_{+,i}(t) + \beta_{\text{soc}-} O_{-,i}(t) + \text{B}_i\big), \qquad (3)$$

where $N_{+,i}(t)$ is the number of times a choosing individual had visited the palatable feeder (positive personal information), $N_{-,i}(t)$ is the number of times a choosing individual had visited the unpalatable feeder (negative personal information), $O_{+,i}(t)$ is the expected number of observed positive (positive social information) and $O_{-,i}(t)$ observed negative feeding events (negative

social information). Bird identity was included as a random effect, B_i (age and species were later added as variables, see Methods in the main text). Parameters $\beta_{\text{asoc}+}$ and $\beta_{\text{asoc}-}$ are the effects of asocial learning about the palatable and unpalatable foods, $\beta_{\text{soc}+}$is the effect of social learning about the palatable food and $\beta_{\text{soc}-}$is the effect of social avoidance learning about the unpalatable food.

We examined the Pearson residuals from the model to assess the model assumptions, first plotting the residuals against the value of each predictor variable to assess whether the effects of each were linear on the log odds scale. Since the response variable is binary, this resulted in a banded pattern of residuals which is difficult to interpret. We therefore divided each predictor into a number of intervals and took the mean of residuals within each range allowing us to detect any trend in the residuals (see the R code 'GLMM models Orange Yellow final.r' in Supplementary data[1]). In all cases, we found that the average residual stayed constant across the range of each predictor variable, suggesting that a linear relationship on the log-odds scales is at least a reasonable approximation for the effect of these variables.

Second, we analysed the residuals to test for autocorrelation in choice of palatable/ unpalatable within each bird, i.e. the possibility that choices made close together in time were more likely to be the same that predicted by the model. We used a linear mixed effects model using the *nlme* package[2], with residual as the response variable, bird identity as a random effect, and choices within each bird correlated as a function of how close together they were in time (corCAR1 function, see the R code 'GLMM models Orange Yellow final.r' in Supplementary data[1]). This yielded an estimate of how strong the autocorrelation was as a function of time difference (phi parameter, correlation = time difference ^ phi). We used the phi parameter to estimate how far apart in time choices needed to be to be considered independent (< 0.01 correlation), giving us a threshold of 15 secs (yellow/orange); 24 secs (blue/purple); 34 secs (red/green). We then cut out choices that were less than the threshold time since the previous choice by that bird (yellow/orange 9%; blue/purple 9%; red/green 13%), and refitted the model. We saw some reduction in apparent statistical power, as expected by a smaller sample size, but the results were otherwise unchanged in each case, suggesting our findings were robust to inclusion/ exclusion of the autocorrelated data. We present the results of the reduced dataset in the main text, and used the reduced dataset for further analysis.

By using the expected number of observations of negative feeding events, $O_{-,i}(t)$, as a measure of the real number of observations, $R_{-,i}(t)$, we were ignoring our uncertainty in the value of $R_{-,i}(t)$. To illustrate this, imagine i had a single connection of strength 0.4 to another bird, j, which had fed at the unpalatable feeder $N_{-,j}(t)$ =4 times, and we assumed $p_o = 1$. We derived $O_{-,i}(t) = N_{-,j}(t)a_{ij}$ = 4 × 0.4 = 1.6. In our model we treated $O_{-,i}(t) = 1.6$ as if it was the real number of observations, but in reality there is a probability distribution for the number of times i had observed negative events, assuming independence of events: p(0) = 0.1296, p(1) = 0.3456, p(2) = 0.3456, p(3) = 0.1536, p(4) = 0.0256. Ideally, we would take this uncertainty into account in the model. However, this is computationally infeasible as each bird had connections of different strengths to many other birds each with different values of $N_{-,j}(t)$. Instead, we ran simulations to test the validity of our approach, testing whether the type 1 error was appropriate and whether a real effect could be detected despite ignoring this source of uncertainty.

Furthermore, the nature of the experiment means that all the predictor variables are inevitably correlated since $N_{-,i}(t)$, $N_{+,i}(t)$, $O_{-,i}(t)$ and $O_{+,i}(t)$ will always increase over time. We were unable to drop or combine any predictors to reduce collinearity, since our aim was to estimate the social learning effects once asocial learning effects had been statistically controlled for. This means there is a potential risk for the effects of one variable to be obscured by the presence of another, or for effects of one variable to be misidentified as the effects of another variable. Consequently, we had two reasons to test whether our modelling approach could reliably detect and estimate the social learning effects of interest. Here we first report and discuss the simulations for the yellow/orange experiment. The results for red/green and blue/purple experiments were very similar (see below).

In our simulations, for each feeding event we randomly determined whether each bird in the population observed that event with a probability equal to the network connection with the feeding bird. Thus, we could track the simulated 'real' number of observations of each type that each bird had $R_{\mathrm{SIM}\,-,i}(t)$ and $R_{\mathrm{SIM}+,i}(t)$ alongside the simulated $N_{\mathrm{SIM}+,i}(t)$ and $N_{\mathrm{SIM}-,i}(t)$. We then simulated the choice (unpalatable versus palatable) for each feeding event (with the identity of the feeder taken from the real data), with the probability of choosing unpalatable determined by the model described in Equation (3), but with $O_{-,i}(t)$ replaced with $R_{\mathrm{SIM}-,i}(t)$ and $O_{+,i}(t)$ with $R_{\mathrm{SIM}+,i}(t)$. The asocial effects $\beta_{\mathrm{asoc}+}$ and $\beta_{\mathrm{asoc}-}$ were taken from a model including only asocial learning effects fitted to the real data, to obtain realistic effect sizes. The simulations were run with various values of $\beta_{\mathrm{soc}+}$and β_{soc-} (see below). Once we had generated the simulated dataset, we calculated $O_{\mathrm{SIM}-,i}(t)$ and $O_{\mathrm{SIM}+,i}(t)$ using Equations (1-2) replacing $N_{+,i}(t)$ and $N_{-,i}(t)$ with $N_{\mathrm{SIM}+,i}(t)$ and $N_{\mathrm{SIM}-,i}(t)$. We then fitted the model in Equation (3) using $N_{\mathrm{SIM}+,i}(t)$, $N_{\mathrm{SIM}-,i}(t)$, $O_{\mathrm{SIM}+,i}(t)$ and $O_{\mathrm{SIM}-,i}(t)$ as predictors, and recorded whether the different social effects were detected.

We repeated this process for 1000 simulated datasets for different combinations of parameter values. We ran simulations with $\beta_{soc+} = 0$ and $\beta_{\mathrm{soc}-} = \{-0, -0.02, -0.04, -0.06, -0.08, -0.1\}$ to test a) whether the type 1 error rate was appropriate for $\beta_{\mathrm{soc}-} = 0$; b) whether statistical power to detect $\beta_{\mathrm{soc}-}$ increased as $\beta_{\mathrm{soc}-}$ increased; c) whether there was any bias in estimates of $\beta_{\mathrm{soc}-}$; d) how often 95% Wald confidence intervals contained the true simulated value of $\beta_{\mathrm{soc}-}$ or tended to overestimate $\beta_{\mathrm{soc}-}$. We then ran simulations with $\beta_{\mathrm{soc}-} = 0$ varying the value of $\beta_{\mathrm{soc}+}$. Since there tended to be many more feeds at the palatable feeder than at the unpalatable feeder, the values of $\beta_{\mathrm{soc}+}$ were chosen to be equivalent in standardized effect size to the values considered for $\beta_{\mathrm{soc}-}$. An example of the R code used to run these simulations can be found in Supplementary data[1] in 'Simulations to Test Methods Network Orange Yellow.r'.

We found that when $\beta_{\mathrm{soc}-}$ and $\beta_{\mathrm{soc}+}$ were set to zero in the simulations an effect of $\beta_{\mathrm{soc}-}$ was detected ($p < 0.05$) 8.9% of the time. However, this was due to an inflated type 1 error rate in the *opposite* direction to that expected for social avoidance learning, i.e. $\beta_{\mathrm{soc}-} > 0$. If we look at the type one errors resulting in a spurious social avoidance learning event, i.e. $p < 0.05$ and $\beta_{\mathrm{soc}-}$ estimated at < 0, we get 0.0% (0/1000 simulations, see Supplementary Table 7). This error rate remained below 2.5% as required as the value of $\beta_{\mathrm{soc}+}$was increased, indicating that the presence of social appetitive learning did not increase the risk of a spurious social avoidance learning effect being detected (Supplementary Table 8). As the value of $\beta_{\mathrm{soc}-}$ increased, the power to detect an effect of $\beta_{\mathrm{soc}-} < 0$ increased as expected (Supplementary Table 7). The mean value for $\beta_{\mathrm{soc}-}$

estimated across simulations was found to be correlated with, but slightly less negative than its true value in the simulations (Supplementary Table 7). The Wald 95% confidence intervals (C.I.) for $\beta_{\text{soc}-}$ were found to contain the true value of $\beta_{\text{soc}-}$ 84-90% of the time, lower than the ideal 95%, however, in cases where $\beta_{\text{soc}-}$was outside the 95% C.I. it was almost always underestimated in magnitude (i.e. the C.I.s were closer to zero than the true value of $\beta_{\text{soc}-}$, see Supplementary Table 7). These results suggest that our method is a likely to be a conservative estimate of the effect of social avoidance learning. Overall, the simulations showed that our method can reliably detect a social avoidance learning effect whereby each observation of another individual feeding on the unpalatable option reduces the probability of the observer choosing the unpalatable option in the future. Furthermore, a positive result for social avoidance learning can be trusted as not being a spurious artifact and indeed estimates of $\beta_{\text{soc}-}$can be viewed as a conservative estimate of its effects.

In contrast, there was an inflated type 1 error for a spurious effect of social appetitive learning ($p < 0.05$ and $\beta_{\text{soc}+}$ estimated at < 0) of around 12.4% (Supplementary Table 7). The mean estimate of $\beta_{\text{soc}+}$ also tended to be slightly more negative than its true value in the simulation with 95% C.I.s that also tended to over-estimate the effect (Supplementary Table 8). We suspect this occurred because opportunities to observe positive feeding events were relatively common (compared to negative feeding events), thus $O_{+,i}(t)$ was more highly correlated with $N_{+,i}(t)$, meaning a spurious effect of the former could be detected as a byproduct of asocial learning about the positive option. This means that estimates of the effects of social appetitive learning should be considered to be anti-conservative (more negative). In practice we suggest that a social appetitive learning only be inferred when there is strong evidence that the effect follows the network (see the main text). Since our primary goal was to detect social avoidance learning these results did not greatly affect our conclusions. Note that in our yellow/orange experiment, we detected a *positive* heterospecific effect of $O_{+,i}(t)$ (see Table 1 in the main text): this is unlikely to be a spurious statistical effect since $\beta_{\text{soc}+}$tended to be estimated as more negative than its true value. We repeated the simulations for red/green and blue/purple experiments. In these cases, we considered an extended range of effect sizes of $\beta_{\text{soc}-}$ to reflect both the decreased sample size in the red/green and blue/purple experiments. We obtained similar results as for the yellow/orange (Supplementary Tables 9-12), with results indicating a conservative estimate of the social avoidance learning. Again, we see an inflated probability of spurious social appetitive learning, indeed this problem is exacerbated in these diffusions, supporting our conclusion that a social appetitive learning only be inferred when there is strong evidence that the effect follows the network.

Robustness to exclusion of network data

In our primary analysis we used networks constructed from data collected outside learning experiments when birds were presented with plain almonds (in total 92 days between 5 June and 17 Sep 2018). It is possible that using only network data collected during a time period closer to each experiment could provide a more accurate network. However, this comes with the trade-off of a reduced sample size and potentially less accurate network. We investigated this issue for the primary orange/yellow experiment, by considering different thresholds for inclusion of network data before and after the start of the experiment, days before: 0, 5, 10, 15, 30, 60, 91 (= all data before), days after: 0, 5, 10, 25 (= all data after). We considered every combination of these thresholds in the analyses described below.

In each case we reconstructed the network, and calculated the correlation with the network constructed from the full data. As expected, in general the correlation decreased as more data was dropped (Supplementary Table 13). However, we would expect the correlation to decrease with a reduction in data even if the underlying network was unchanging. To assess what correlation we would expect from a reduction in sample size alone, we randomly resampled the network data without replacement for a range of sample sizes and calculated the correlation with the full data network. We repeated this 100x for each sample size we considered, and calculated the mean and central 95% (see Supplementary Figure 5 and the R code 'Robustness to exclusion of network data.r' in Supplementary data[1]).

Correlation with the full network was lowest for the network constructed only from data collected within the learning experiment (days when the experiment was halted for mist netting and ringing sessions and birds were presented with plain almonds). However, this was within the range expected purely from a reduction in sample size to 1027 data points. We therefore concluded this network was likely to be highly inaccurate, and decided not to use this network for our main analysis. In other cases, the correlation was lower than expected due to a reduction in sample size alone, supporting the notion that the network was changing over time. In most cases, the correlation remained high suggesting that the network was changing slowly, and that the full network might be a good approximation to a smaller time period. However, the next lowest correlations are observed when we dropped the network data collected after the experiment, suggesting the experiment itself may have induced some changes in the network structure. Consequently, to ensure that this was not driving the effects in the model, we conducted further analyses to check that the main analysis is robust to the network data that is included in the social network.

For each network displayed in Supplementary Table 13, we re-ran the analysis and assessed the effect on the model parameters and their significance. To the p-value for evidence of following the network we used the same null distributions as for the main analysis since the simulations used to generate these should be approximately applicable across networks (and re-running the simulations for all 28 networks was computationally infeasible). The effect on the model estimates is shown in Supplementary Tables 14-18. Overall, the analysis was very robust to inclusion/exclusion of data from before and after the experiment was conducted. The direction of effects was consistent as was the pattern of significance. The magnitude of effects was very similar in all cases except for the effect of conspecifics feeding on palatable almonds: here there was some variation in relative magnitude, but this effect was nonetheless always estimated as small and statistically non-significant. To avoid making an arbitrary decision on the amount of network data to include, we report the analysis based on the full data network in the main text.

Assortativity analysis

Assortativity is a phenomenon that characterizes the preferential interaction between individuals with similar characteristics such as sex or age. Assortativity values range from -1 (total disassortativity) to 1 (total assortativity). In our experiment, we found that the learning rate from adults was higher compared to the learning rate from juveniles, which could be related to the fact that juveniles spend more time with adults than with other juveniles. Thus, we expected to observe disassortativity or a small value of assortativity. In order to determine whether the network showed evidence of assortativity, we performed data stream permutations in order to control for feeders' location and the different time windows that defined individuals' associations, to build a null

model with 10000 permutations. The analysis was conducted using *ANTs* package[3]. In accordance with our predictions, the results showed the presence of a very low value of assortativity (0.10), which was lower than expected by chance ($P < 0.001$, see Supplementary Table 19 and Supplementary Figure 6 for more details). This result suggests that individuals tended to associate without age discrimination and such phenomenon could be the proximal mechanism that allowed juveniles to learn from adults.

Reversal learning: Supplementary results

The best-supported NBDA models included social transmission following our observed network (Table 3 in the main text). There was no strong evidence for different asocial learning rates between the two species: blue tits were estimated to be slightly faster at sampling blue almonds than great tits (estimated effect of species (blue tit) = 1.48x faster), but also opposite effect was possible (95 % CI: 0.83–2.61). Similarly, we did not find strong support for differences in social learning: great tits were estimated to have a faster social learning rate than blue tits (estimated effect of species (great tit) = 2.10x faster), but also equal learning rates were possible (95 % CI: 0.99–4.41).

The estimated social transmission parameters in the best-fit model were 10.83 (95 % CI: 1.60–67.26) from adults and 0 (95 % CI: 0–1.75) from juveniles, suggesting that an observation of adults feeding on blue almonds had a stronger effect on observers' decisions to sample the same colour (95 % CI for the difference in social transmission rates from adults and juveniles: 1.60–67.3). We further investigated potential differences in social transmission between conspecifics and heterospecifics. Because social transmission happened mainly by observing adults, we investigated this by fitting a model in which we assumed social transmission only from adults, and which included different conspecific and heterospecific transmission rates, and different asocial and social learning rates between the two species. The estimated social transmission parameters were 14.47 (95 % CI: 2.02–98.52) between conspecifics and 6.84 (95 % CI: 0.78–53.29) between heterospecifics, suggesting that there was social transmission both within and between the species. There was no clear evidence of differences in the strength of social transmission between conspecifics and heterospecifics, with potential differences possible in either direction (95 % CI for the difference in social transmission rates from conspecifics and heterospecifics: -17.79–65.86).

2. Supplementary Figures

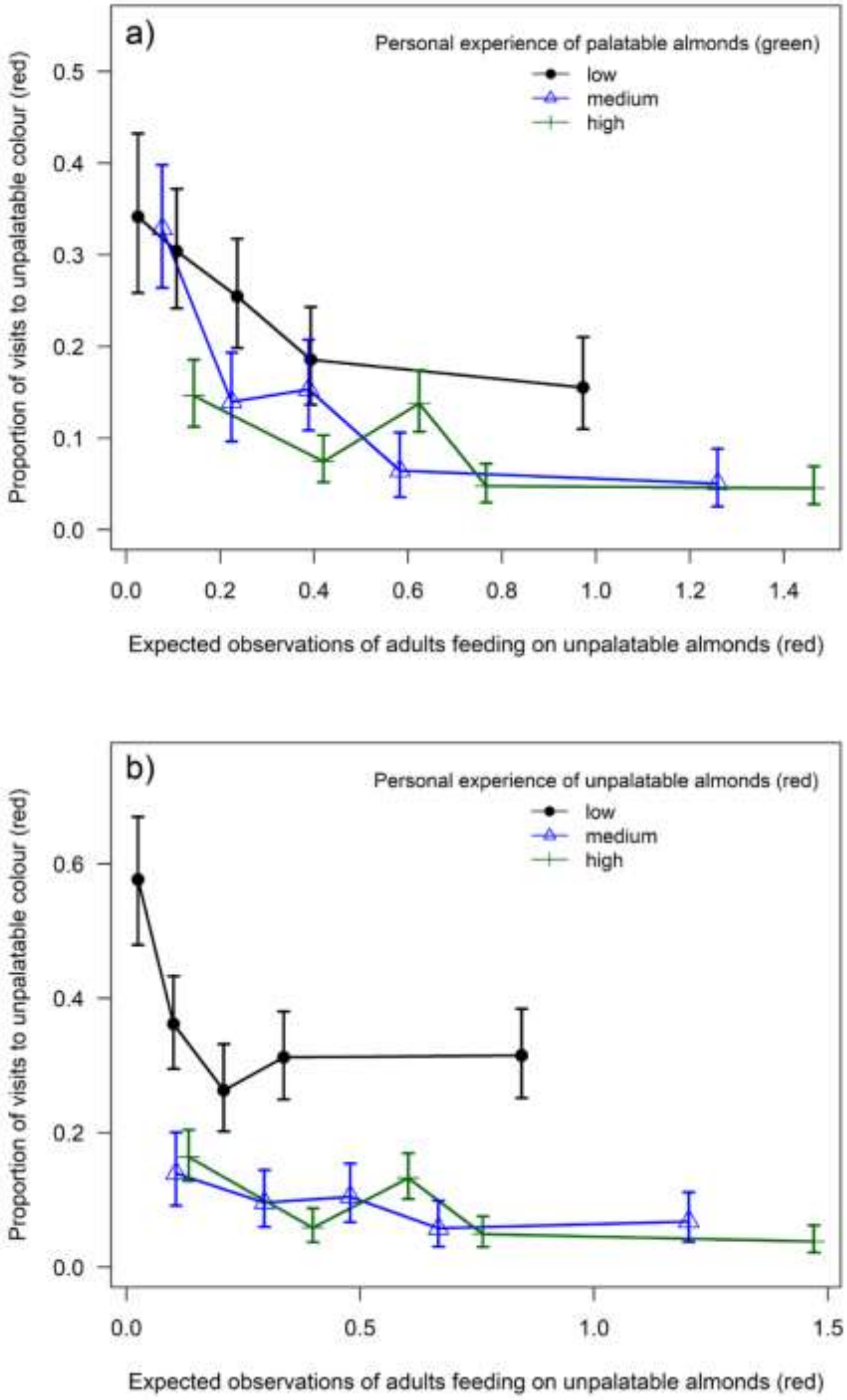

Supplementary Figure 1. Proportion of birds (n = 86) choosing the unpalatable (red) option in the red/green experiment after observing adults feeding on unpalatable almonds. Social information reduced the likelihood to choose the unpalatable colour when birds had little personal experience of (a) palatable or (b) unpalatable almonds (circles and black lines). For illustration

purposes, the data is divided into ‘personal experience categories’ (represented by different symbols and colours), based on how many times birds had personally sampled palatable (a) or unpalatable (b) almonds before their current choice, standardized within each bird to allow us to show the within-bird patterns detected by the model. ‘Low asocial experience’ includes data from birds within the 1st quartile for this variable, ‘medium’ birds within the 2nd quartile, and ‘high’ birds within the 3rd or 4th quartile. Within these ‘personal experience categories’, the data is further split into categories based on the expected number of observed unpalatable feeding events of adults. Symbols show the mean and 95 % CI for the proportion of birds choosing the unpalatable option.

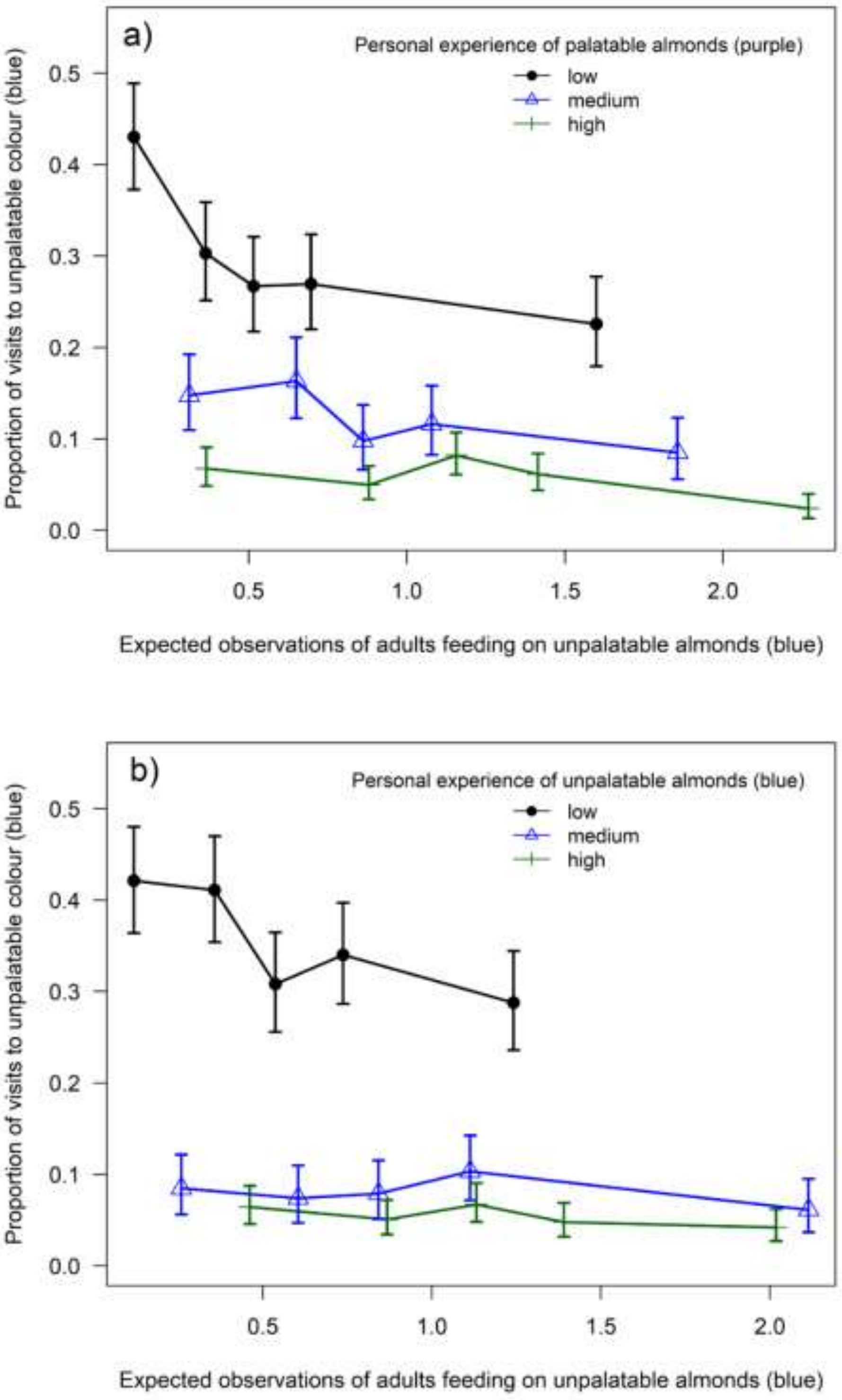

Supplementary Figure 2. Proportion of birds (n = 90) choosing the unpalatable (blue) option in the blue/purple experiment after observing adults feeding on unpalatable almonds. Social information reduced the likelihood to choose the unpalatable colour when birds had little personal experience of (a) palatable or (b) unpalatable almonds (circles and black lines). For illustration

purposes, the data is divided into personal experience categories' (represented by different symbols and colours), based on how many times birds had personally sampled palatable (a) or unpalatable (b) almonds before their current choice, standardized within each bird to allow us to show the within-bird patterns detected by the model. 'Low asocial experience' includes data from birds within the 1st quartile for this variable, 'medium' birds within the 2nd quartile, and 'high' birds within the 3rd or 4th quartile. Within these 'personal experience categories', the data is further split into categories based on the expected number of observed unpalatable feeding events of adults. Symbols show the mean and 95 % CI for the proportion of birds choosing the unpalatable option.

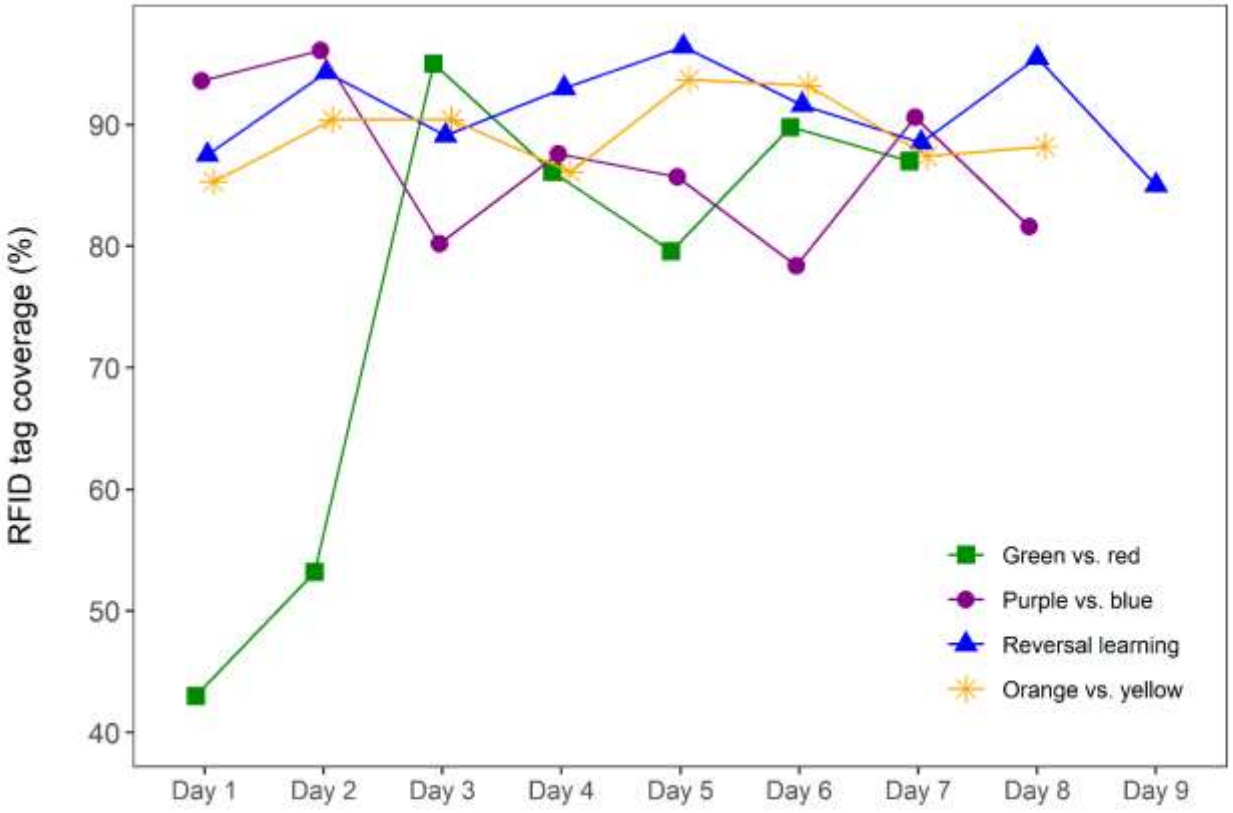

Supplementary Figure 3. RFID tag coverage (%) across days in each experiment (number of visiting blue tits and great tits that had RFID tags, divided by all visiting blue tits and great tits). In the first experiment (red/green) the RFID tag coverage was low during the first two days and we conducted a mist netting session before continuing the experiment. For the other colour pairs, we conducted a mist netting session four (reversal learning) or five days (blue/purple and yellow/orange) after the experiment was started to maintain a high RFID tag coverage (see Methods in the main text).

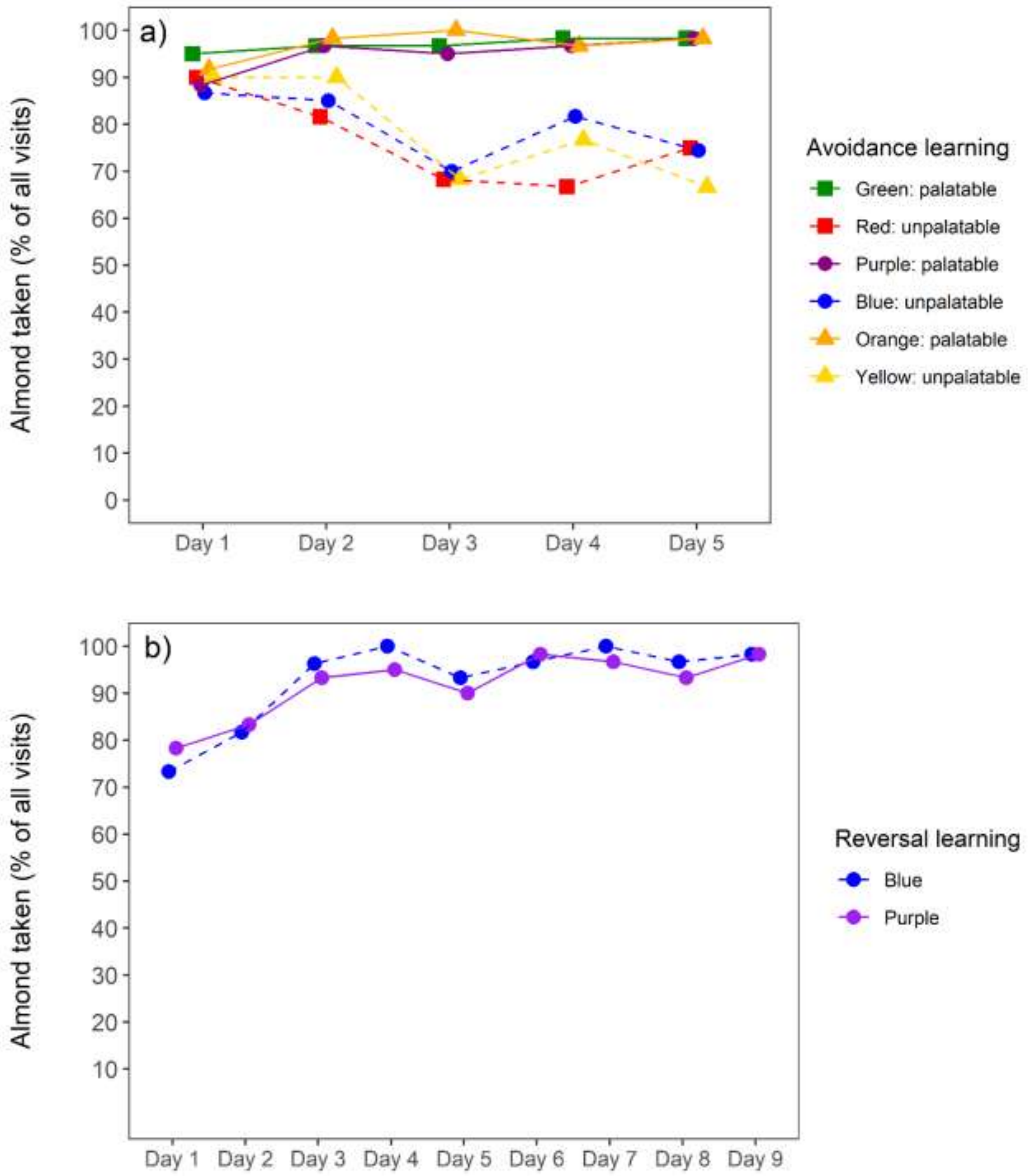

Supplementary Figure 4. Percentage of visits that included a feeding event (birds ate or took the almond) during (a) avoidance and (b) reversal learning experiments (see Methods in the main text).

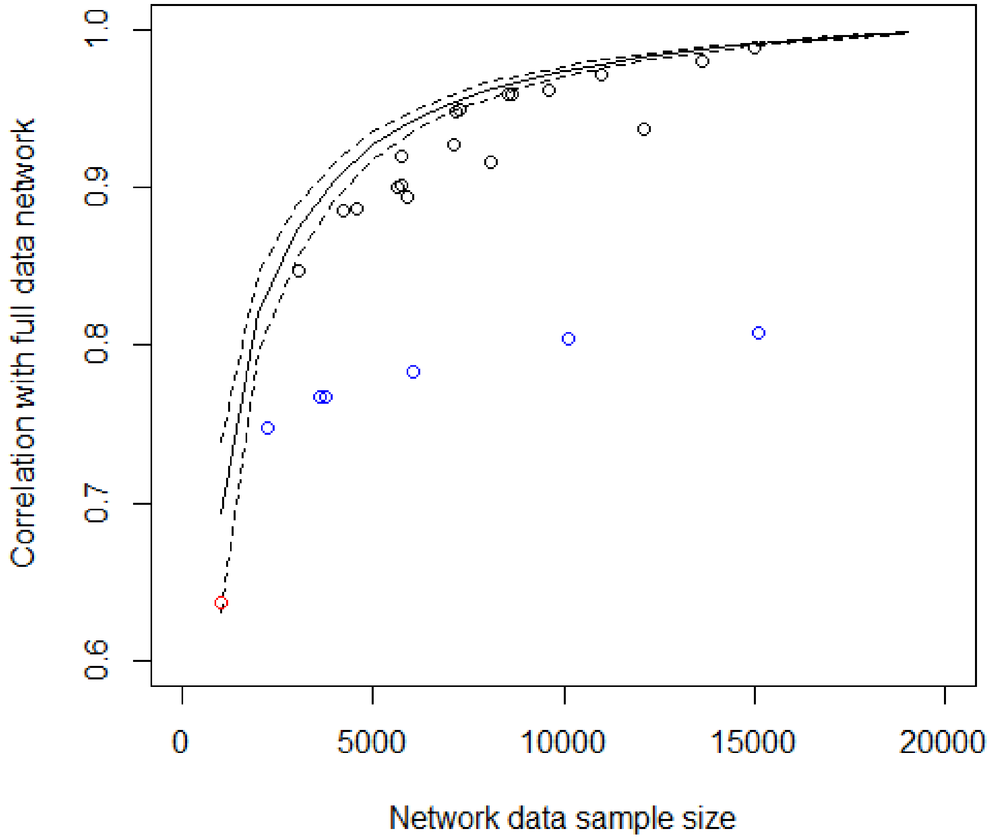

Supplementary Figure 5. Correlation of cut data networks with full data network plotted against sample size. The line shows the expected correlation based on a reduction in sample size alone, with 95% prediction intervals (dashed lines). The red point shows the network constructed from data collected only during the experiment, the blue points show networks constructed from data collected only before the experiment

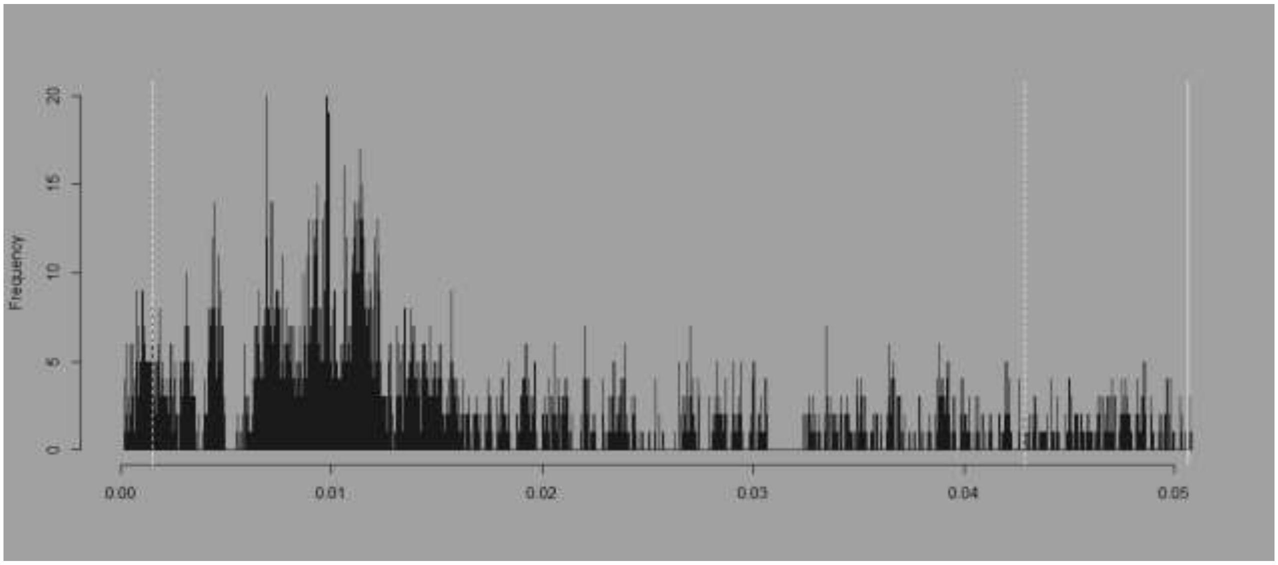

Supplementary Figure 6. Results of assortativity data stream permutations (posterior distribution). Dashed white lines represent the confidence intervals and the plain white line represents the assortativity value from the observed network.

3. Supplementary Tables

Supplementary Table 1. Best-fit generalized linear mixed effects model explaining birds' foraging choices during avoidance learning experiments (across 8 days) and the comparison of models.
A) Summary of the best-fitting GLMM model. Birds' (n = 189) choices were modelled using a binomial error distribution, with the number of visits to palatable and unpalatable feeders as a bound response variable, and this was explained by the interaction between individuals' age and the day of the experiment (a second order polynomial term). Bird identity (variance = 0.501) was included as a random effect.
B) Comparison of GLMMs explaining birds' foraging choices. Abbreviations of the explanatory variables are: A = age, S = species, D = day, ID = bird identity. We started a model selection with a model that included a three-way interaction between the species, age and day (a second order polynomial term), and selected the best-fit model based on Akaike's information criterion.

A) Final model

Terms in the model	Estimate	SE	Z	P
Intercept	-2.501	0.138	-18.190	< 0.0001
Age (juvenile)	0.474	0.154	3.087	0.002
Day (linear)	-42.527	3.583	-11.869	< 0.0001
Day (polynomial)	32.694	3.344	9.776	< 0.0001
Day (linear) * Age (juvenile)	5.296	3.853	1.374	0.17
Day (polynomial) * Age (juvenile)	-17.734	3.593	-4.935	< 0.0001

B) Model selection

Alternative models	AIC	ΔAIC
~A*D (poly) + A*D (linear) + 1|ID (final model)	5990.6	0
~A*D (poly) + A*D (linear) + S + 1|ID	5991.2	+0.6
~A*D (poly) + A*D (linear) + S*D (poly) + S*D (linear) + 1|ID	5993.7	+3.1
~A*S*D (poly) + A*S*D (linear) + 1|ID	5994.0	+3.4
~A*D (poly) + A*D (linear) + S*D (poly) + S*D (linear) + A*S + 1|ID	5995.6	+5.0
~A*D (linear) + 1|ID (best model with linear terms only)	6197.6	+207.0

Supplementary Table 2. Best-fit generalized linear mixed effects model explaining birds' foraging choices during reversal learning experiment (across 9 days) and the comparison of models.

A) Summary of the best-fitting GLMM model. Birds' (n = 118) choices were modelled using a binomial error distribution, with the number of visits to purple and blue feeders as a bound response variable, and this was explained by the interactions between species and the day of the experiment (linear term), and individuals' age and the day of the experiment (a second order polynomial term). Bird identity (variance = 1.603) was included as random effect.

B) Comparison of GLMMs explaining birds' foraging choices. Abbreviations of the explanatory variables are: A = age, S = species, D = day, ID = bird identity. We started a model selection with a model that included a three-way interaction between the species, age and day (a second order polynomial term), and selected the best-fit model based on Akaike's information criterion.

A) Final model

Terms in the model	Estimate	SE	Z	P
Intercept	-2.561	0.347	-7.383	< 0.001
Age (juvenile)	0.030	0.260	0.116	0.91
Species (great tit)	0.515	0.331	1.558	0.12
Day (linear)	0.262	0.028	9.382	< 0.0001
Day (polynomial)	-5.094	1.517	-3.357	0.0008
Day (linear) * Age (juvenile)	0.195	1.928	0.101	0.92
Day (polynomial) * Age (juvenile)	5.496	1.754	3.133	0.002
Day (linear) * Species (great tit)	-0.045	0.025	-1.785	0.074

B) Model selection

Alternative models	AIC	ΔAIC
~A*D (poly) + A*D (linear) + S*D (linear) + 1|ID (final model)	4669.5	0
~A*D (poly) + + A*D (linear) + S*D (poly) + S*D (linear) + 1|ID	4671.5	+2.0
~A*S*D (poly) + A*S*D (linear) + 1|ID	4672.6	+3.1
~A + S + D + 1|ID (best model with linear terms only)	4673.3	+6.8

Supplementary Table 3: Best-fit generalized linear mixed effects model explaining the effect of personal and social information on birds' foraging choices in the red/green experiment and the comparison of models.

A) Summary of the best-fitting GLMM model. Birds' (n = 86) choices were modelled using a binomial error distribution, with each choice as a binary response variable. An intercept gives a likelihood to choose an unpalatable colour (red). This was explained by birds' previous visits to the palatable (green) and unpalatable (red) feeder, as well as observations of negative and positive feeding events of others, split between observations of adults and juveniles. Observations of positive feeding events were further divided to observations of conspecifics (CS) and heterospecifics (HS). Bird identity (variance = 3.067) was included as a random effect. Coefficients give an estimate of the effect of one visit or observation on the likelihood to choose an unpalatable option.

B) Comparison of GLMMs explaining birds' foraging choices in the red/green experiment.

A) Final model

**Terms in the model**	**Estimate**	**SE**	**Z**	**P**
Intercept	-0.187	0.041	-4.545	< 0.0001
Visit to palatable feeder (green)	-0.092	0.029	-3.162	0.002
Visit to unpalatable feeder (red)	-0.071	0.028	-2.538	0.011
Positive observation of CS juvenile	0.017	0.012	1.430	0.15
Positive observation of CS adult	-0.089	0.055	-1.612	0.11
Positive observation of HS juvenile	0.030	0.011	2.720	0.007
Positive observation of HS adult	-0.090	0.102	-0.878	0.38
Negative observation of juvenile	-0.086	0.047	-1.838	0.066
Negative observation of adult	-0.774	0.598	-1.294	0.20

B) Model selection

**Alternative models**	**AIC**	**ΔAIC**
Final model (Supplementary Table 3A)	2433.2	0
Different conspecific/heterospecific positive effect, no age differences in social effects	2441.9	+8.7
Different conspecific/heterospecifics social effects, no age differences in social effects	2443.7	+10.5
No conspecific/heterospecific or age differences in social effects	2441.2	+8.0

Supplementary Table 4. Best-fit generalized linear mixed effects model explaining the effect of personal and social information on birds' foraging choices in the blue/purple experiment and the comparison of models.

A) Summary of the best-fitting GLMM model. Birds' (n = 90) choices were modelled using a binomial error distribution, with each choice as a binary response variable. An intercept gives a likelihood to choose an unpalatable colour (blue). This was explained by birds' previous visits to the palatable (purple) and unpalatable (blue) feeder, as well as observations of negative and positive feeding events of others, split between observations of adults and juveniles. Observations of positive feeding events were further divided to observations of conspecifics (CS) and heterospecifics (HS). Bird identity (variance = 1.817) was included as a random effect. Coefficients give an estimate of the effect of one visit or observation on the likelihood to choose an unpalatable option.

B) Comparison of GLMMs explaining birds' foraging choices in the blue/purple experiment.

A) Final model

**Terms in the model**	**Estimate**	**SE**	**Z**	**P**
Intercept	-0.007	0.031	-0.227	0.82
Visit to palatable feeder (purple)	-0.047	0.021	-2.209	0.027
Visit to unpalatable feeder (blue)	-0.091	0.017	-5.223	< 0.0001
Positive observation of CS juvenile	0.003	0.008	0.306	0.76
Positive observation of CS adult	-0.114	0.049	-2.311	0.021
Positive observation of HS juvenile	0.024	0.010	2.417	0.016
Positive observation of HS adult	0.017	0.060	0.278	0.78
Negative observation of juvenile	-0.060	0.038	-1.556	0.12
Negative observation of adult	-0.656	0.439	-1.493	0.14

B) Model selection

**Alternative models**	**AIC**	**ΔAIC**
Final model (Supplementary Table 4A)	3266.7	0
Different conspecific/heterospecific positive effect, no age differences in social effects	3279.5	+12.8
Different conspecific/heterospecifics social effects, no age differences in social effects	3276.3	+9.6
No conspecific/heterospecific or age differences in social effects	3278.3	+11.6

Supplementary Table 5. Best-fit generalized linear mixed effects model explaining the effect of personal and social information on birds' foraging choices in the yellow/orange experiment and the comparison of models.
A) Summary of the best-fitting GLMM model. Birds' (n = 168) choices were modelled using a binomial error distribution, with each choice as a binary response variable. An intercept gives a likelihood to choose an unpalatable colour (yellow). This was explained by birds' previous visits to the palatable (orange) and unpalatable (yellow) feeder, as well as observations of negative and positive feeding events of others, split between observations of adults and juveniles. Observations of positive feeding events were further divided to observations of conspecifics (CS) and heterospecifics (HS). Bird identity (variance = 0.812) was included as a random effect. Coefficients give an estimate of the effect of one visit or observation on the likelihood to choose an unpalatable option.
B) Comparison of GLMMs explaining birds' foraging choices in the yellow/orange experiment.

A) Final model

**Terms in the model**	**Estimate**	**SE**	**Z**	**P**
Intercept	-0.011	0.005	-2.046	0.041
Visit to palatable feeder (orange)	-0.022	0.006	-3.854	0.0001
Visit to unpalatable feeder (yellow)	-0.014	0.004	-3.576	0.0003
Positive observation of CS juvenile	0.002	0.003	0.814	0.42
Positive observation of CS adult	-0.053	0.013	-4.080	< 0.0001
Positive observation of HS juvenile	0.018	0.003	5.568	< 0.0001
Positive observation of HS adult	-0.051	0.014	-3.724	0.0002
Negative observation of juvenile	-0.006	0.015	-0.425	0.67
Negative observation of adult	-0.381	0.112	-3.408	0.0007

B) Model selection

**Alternative models**	**AIC**	**ΔAIC**
Final model (Supplementary Table 5A)	9272.4	0
Different conspecific/heterospecific positive effect, no age differences in social effects	9370.0	+97.6
Different conspecific/heterospecifics social effects, no age differences in social effects	9371.3	+98.9
No conspecific/heterospecific or age differences in social effects	9381.20	+108.8

Supplementary Table 6. Comparison of final GLMMs including both social and asocial effects (Supplementary Tables 3-5) to models where either asocial or social effects were dropped (dropping both social effects or only positive/negative).

	**Effect on AIC**		
**Drop effect:**	**Red/Green**	**Blue/Purple**	**Yellow/Orange**
**Both asocial effects**	+42.5	+82.3	+71.3
**Both social effects**	+6.90	+8.78	+80.5
**Observing a negative feeding experience:**	+1.84	+1.95	+37.8
**Observing a positive feeding experience:**	-1.64	-3.00	+12.1

Supplementary Table 7. Results of simulations (1000 per set of parameter values) for the yellow/orange experiment as the value of $\beta_{\text{soc}-}$ was varied ($\beta_{\text{soc}+} = 0$ in all simulations). See supplementary text above for explanation.

	**True value of $\boldsymbol{\beta}_{\textbf{soc}-}$ in simulations (per observation)**					
	0	-0.02	-0.04	-0.06	-0.08	-0.10
**Power to detect social avoidance learning $\boldsymbol{\beta}_{\textbf{soc}-} < 0$ (%)**	0.0	32.5	88.1	99.2	99.9	100.0
**Mean estimate of $\boldsymbol{\beta}_{\textbf{soc}-}$**	0.005	-0.013	-0.033	-0.051	-0.071	-0.090
**95% C.I. coverage for $\boldsymbol{\beta}_{soc-}$ (%)**	91.0	90.5	91.2	91.9	90.5	92.6
**95% C.I. overestimate of effect size for $\boldsymbol{\beta}_{\textbf{soc}-}$(%)***	0.0	0.0	0.2	0.0	0.2	0.2
**Type 1 error for social appetitive learning $\boldsymbol{\beta}_{\textbf{soc}+} < 0$ (%)**	12.3	11.8	12.7	10.1	10.3	8.3

*True value of $\beta_{\text{soc}-}$closer to zero than the upper (less negative) limit of the confidence interval.

Supplementary Table 8. Results of simulations (1000 per set of parameter values) for the yellow/orange experiment as the value of $\beta_{\text{soc}+}$ was varied ($\beta_{\text{soc}-} = 0$ in all simulations). See supplementary text above for explanation.

	**True value of $\boldsymbol{\beta}_{\text{soc}+}$ in simulations (per observation)**					
	0	-0.003	-0.006	-0.009	-0.012	-0.015
**Power to detect social appetitive learning $\boldsymbol{\beta}_{\text{soc}+} < 0$ (%)**	12.8	95.5	100.0	100.0	100.0	100.0
**Mean estimate of $\boldsymbol{\beta}_{\text{soc}+}$**	-0.001	-0.004	-0.007	-0.010	-0.014	-0.017
**95% C.I. coverage for $\boldsymbol{\beta}_{\text{soc}+}$ (%)**	87.2	85.3	88.1	87.4	85.0	85.9
**95% C.I. overestimate of effect size for $\boldsymbol{\beta}_{\text{soc}+}$(%)***	12.7	14.6	11.8	12.5	15.0	13.7
**Type 1 error for social avoidance learning $\boldsymbol{\beta}_{\text{soc}-} < 0$ (%)**	0.1	0.2	0.2	0.1	0.1	0.9

*True value of $\beta_{\text{soc}-}$closer to zero than the upper (less negative) limit of the confidence interval.

Supplementary Table 9. Results of simulations (1000 per set of parameter values) for the red/green experiment as the value of $\beta_{\text{soc}-}$ was varied ($\beta_{\text{soc}+} = 0$ in all simulations). See supplementary text above for explanation.

	**True value of $\boldsymbol{\beta}_{\text{soc}-}$ in simulations (per observation)**								
	0	-0.02	-0.04	-0.06	-0.08	-0.10	-0.30	-0.40	-0.50
**Power to detect social avoidance learning $\boldsymbol{\beta}_{\text{soc}-} < 0$ (%)**	0.3	0.8	2.0	3.1	12.1	25.0	27.8	57.6	71.3
**Mean estimate of $\boldsymbol{\beta}_{\text{soc}-}$**	0.017	0.004	-0.015	-0.026	-0.040	-0.056	-0.218	-0.307	-0.373
**95% C.I. coverage for $\boldsymbol{\beta}_{\text{soc}-}$ (%)**	96.8	97.7	96.6	93.8	85.4	72.2	78.5	88.3	84.9
**95% C.I. overestimate of effect size for $\boldsymbol{\beta}_{\text{soc}-}$(%)***	0.3	0.2	0.4	0.4	3.0	5.9	8.3	2.3	0.7
**Type 1 error for social appetitive learning $\boldsymbol{\beta}_{\text{soc}+} < 0$ (%)**	16.2	18.6	19.4	23.1	29.0	39.3	30.7	20.5	16.8

*True value of $\beta_{\text{soc}-}$closer to zero than the upper (less negative) limit of the confidence interval.

Supplementary Table 10. Results of simulations (1000 per set of parameter values) for the red/green experiment as the value of $\beta_{\text{soc}+}$ was varied ($\beta_{\text{soc}-} = 0$ in all simulations). See supplementary text above for explanation.

	True value of $\boldsymbol{\beta_{\text{soc}+}}$ in simulations (per observation)					
	0	-0.003	-0.007	-0.010	-0.014	-0.017
**Power to detect social appetitive learning $\boldsymbol{\beta_{\text{soc}+} < 0}$ (%)**	15.0	29.2	54.1	71.8	88.7	96.3
**Mean estimate of $\boldsymbol{\beta_{\text{soc}+}}$**	-0.009	-0.012	-0.017	-0.020	-0.025	-0.029
**95% C.I. coverage for $\boldsymbol{\beta_{\text{soc}+}}$ (%)**	85.0	82.1	80.1	75.6	62.9	47.1
**95% C.I. overestimate of effect size for $\boldsymbol{\beta_{\text{soc}+}}$(%)***	15.0	17.9	19.9	24.1	35.8	50.9
**Type 1 error for social avoidance learning $\boldsymbol{\beta_{\text{soc}-} < 0}$ (%)**	0.3	0.4	0.3	2.2	6.3	11.1

*True value of $\beta_{\text{soc}-}$closer to zero than the upper (less negative) limit of the confidence interval.

Supplementary Table 11. Results of simulations (1000 per set of parameter values) for the blue/purple experiment as the value of $\beta_{\text{soc}-}$ was varied ($\beta_{\text{soc}+} = 0$ in all simulations). See supplementary text above for explanation.

	True value of $\boldsymbol{\beta_{\text{soc}-}}$ in simulations (per observation)					
	0	-0.05	-0.1	-0.125	-0.15	-0.175
**Power to detect social avoidance learning $\boldsymbol{\beta_{\text{soc}-} < 0}$ (%)**	0.0	6.5	35.6	48.3	68.9	85.8
**Mean estimate of $\boldsymbol{\beta_{\text{soc}-}}$**	0.026	-0.019	-0.067	-0.090	-0.114	-0.141
**95% C.I. coverage for $\boldsymbol{\beta_{\text{soc}-}}$ (%)**	88.9	88.1	89.0	88.4	79.4	57.0
**95% C.I. overestimate of effect size for $\boldsymbol{\beta_{\text{soc}-}}$(%)***	0.0	0.0	0.0	0.8	2.9	9.7
**Type 1 error for social appetitive learning $\boldsymbol{\beta_{\text{soc}+} < 0}$ (%)**	17.8	21.1	20.3	21.7	22.5	20.2

*True value of $\beta_{\text{soc}-}$closer to zero than the upper (less negative) limit of the confidence interval.

Supplementary Table 12. Results of simulations (1000 per set of parameter values) for the blue/purple experiment as the value of $\beta_{\text{soc+}}$ was varied ($\beta_{\text{soc}-} = 0$ in all simulations). See supplementary text above for explanation.

	True value of $\boldsymbol{\beta_{\text{soc+}}}$ in simulations (per observation)					
	0	-0.009	-0.018	-0.022	-0.027	-0.031
**Power to detect social appetitive learning $\boldsymbol{\beta_{\text{soc+}} < 0}$ (%)**	14.9	83.3	99.2	100.0	100.0	100.0
**Mean estimate of $\boldsymbol{\beta_{\text{soc+}}}$**	-0.006	-0.015	-0.024	-0.028	-0.033	-0.037
**95% C.I. coverage for $\boldsymbol{\beta_{\text{soc+}}}$ (%)**	85.0	84.9	84.3	49.1	44.9	78.7
**95% C.I. overestimate of effect size for $\boldsymbol{\beta_{\text{soc+}}}$(%)***	14.9	15.1	15.5	48.3	46.8	17.9
**Type 1 error for social avoidance learning $\boldsymbol{\beta_{\text{soc}-} < 0}$ (%)**	0.0	0.0	1.4	1.4	1.2	3.4

*True value of $\beta_{\text{soc}-}$closer to zero than the upper (less negative) limit of the confidence interval.

Supplementary Table 13. Correlation of cut data networks with the full data network (yellow/orange experiment).

**Days after included**	**Days before included**						
	**0**	**5**	**10**	**15**	**30**	**60**	**91 (all)**
**0**	0.636	0.747	0.767	0.767	0.784	0.804	0.808
**5**	0.848	0.886	0.901	0.902	0.916	0.938	0.944
**10**	0.887	0.921	0.949	0.95	0.962	0.981	0.99
**25 (all)**	0.895	0.927	0.959	0.96	0.972	0.99	1

Supplementary Table 14. Estimates of standardized effect (per SD) of expected observations of feeds on unpalatable almonds when using different amounts of network data (yellow/orange experiment). Cells shaded dark grey (all cases) indicate significance at the 0.1% level in both the GLMM and in the test for whether the effect followed the network.

**Days after included**	**Days before included**						
	**0**	**5**	**10**	**15**	**30**	**60**	**91 (all)**
**0**	-0.757	-0.652	-0.500	-0.499	-0.511	-0.501	-0.459
**5**	-0.682	-0.677	-0.545	-0.545	-0.564	-0.564	-0.527
**10**	-0.754	-0.748	-0.622	-0.623	-0.641	-0.645	-0.610
**25 (all)**	-0.784	-0.774	-0.653	-0.653	-0.671	-0.676	-0.644

Supplementary Table 15. Estimates of standardized effect (per SD) of expected observations of feeds by conspecifics on palatable almonds when using different amounts of network data (yellow/orange experiment). No effects were significant at the 5% level.

Days after included	Days before included						
	0	5	10	15	30	60	91 (all)
0	-0.019	-0.028	-0.028	-0.028	-0.041	-0.047	-0.03
5	-0.060	-0.058	-0.047	-0.047	-0.057	-0.060	-0.046
10	-0.078	-0.078	-0.068	-0.068	-0.077	-0.079	-0.068
25 (all)	-0.069	-0.070	-0.060	-0.06	-0.071	-0.074	-0.065

Supplementary Table 16. Estimates of standardized effect (per SD) of expected observations of feeds by heterospecifics on palatable almonds when using different amounts of network data (yellow/orange experiment). In all cases the effect was significant at the 0.1% level. Cells shaded mid grey indicate significance at the 1% level in the test for whether the effect followed the network, the cell shaded light grey was significant at the 5% level.

Days after included	Days before included						
	0	5	10	15	30	60	91 (all)
0	0.125	0.094	0.141	0.141	0.15	0.154	0.146
5	0.116	0.103	0.118	0.118	0.127	0.132	0.131
10	0.134	0.124	0.122	0.122	0.133	0.139	0.139
25 (all)	0.133	0.124	0.115	0.116	0.128	0.136	0.135

Supplementary Table 17. Estimates of standardized effect (per SD) of previous feeds on palatable almonds when using different amounts of network data (yellow/orange experiment). Cells shaded dark grey (all cases) indicate significance at the 0.1% level.

Days after included	Days before included						
	0	5	10	15	30	60	91 (all)
0	-0.421	-0.494	-0.652	-0.652	-0.636	-0.641	-0.676
5	-0.430	-0.43	-0.574	-0.574	-0.555	-0.555	-0.598
10	-0.355	-0.348	-0.48	-0.479	-0.459	-0.458	-0.506
25 (all)	-0.329	-0.321	-0.445	-0.444	-0.425	-0.422	-0.468

Supplementary Table 18. Estimates of standardized effect (per SD) of previous feeds on unpalatable almonds when using different amounts of network data (yellow/orange experiment). Cells shaded dark grey (all cases) indicate significance at the 0.1% level.

Days after included	Days before included						
	0	5	10	15	30	60	91 (all)
0	-0.570	-0.540	-0.773	-0.772	-0.746	-0.75	-0.803
5	-0.427	-0.411	-0.609	-0.608	-0.585	-0.591	-0.662
10	-0.357	-0.338	-0.483	-0.483	-0.465	-0.476	-0.548
25 (all)	-0.361	-0.340	-0.462	-0.461	-0.442	-0.452	-0.517

Supplementary Table 19. Results of the assortativity analysis (permuted p-values, confidence interval and mean of the posterior distribution). The assortativity value from the observed network was compared to a null distribution build with 1000 data stream permutations.

Assortativity value	p.left	p.right	p.one.side	95ci lower	95ci upper	mean
0.051	0.99	< 0.0001	0.002	0.001	0.042	0.014

4. Supplementary References

1. Hämäläinen, L., Hoppitt, W., Rowland, H.M., Mappes, J., Fulford, A. J., Sosa, S. & Thorogood, R. Data from: Social transmission in the wild reduces predation pressure on novel prey signals. Dryad Digital Repository. https://doi.org/10.5061/dryad.9s4mw6mcv

2. Pinheiro, J., Bates, D., DebRoy, S., Sarkar, D., R Core Team. *nlme: Linear and Nonlinear Mixed Effects Models*. R package version 3.1-150. (2020).

3. Sosa, S., Puga-Gonzalez, I., Hu, F., Pansanel, J., Xie, X., & Sueur, C. A multilevel statistical toolkit to study animal social networks: the animal network toolkit software (Ants) R package. *Sci. Rep*. **10**, 1-8 (2020).
