# Supplementary material for: Social transmission in the wild can reduce predation pressure on novel prey signals
Source: Nat Commun. 2021 Jun 25;12:3978. doi: 10.1038/s41467-021-24154-0 (PMC8233390; doi:10.1038/s41467-021-24154-0)
Supplement: Supplementary file 3 — Reporting Summary [file 41467_2021_24154_MOESM3_ESM.pdf]

nature research

Corresponding author(s): Liisa Hämäläinen

Last updated by author(s): Mar 30, 2021

Reporting Summary

Nature Research wishes to improve the reproducibility of the work that we publish. This form provides structure for consistency and transparency in reporting. For further information on Nature Research policies, see our Editorial Policies and the Editorial Policy Checklist.

Statistics

For all statistical analyses, confirm that the following items are present in the figure legend, table legend, main text, or Methods section.

n/a	Confirmed	
☐	☒	The exact sample size (n) for each experimental group/condition, given as a discrete number and unit of measurement
☐	☒	A statement on whether measurements were taken from distinct samples or whether the same sample was measured repeatedly
☐	☒	The statistical test(s) used AND whether they are one- or two-sided<br>*Only common tests should be described solely by name; describe more complex techniques in the Methods section.*
☐	☒	A description of all covariates tested
☐	☒	A description of any assumptions or corrections, such as tests of normality and adjustment for multiple comparisons
☐	☒	A full description of the statistical parameters including central tendency (e.g. means) or other basic estimates (e.g. regression coefficient) AND variation (e.g. standard deviation) or associated estimates of uncertainty (e.g. confidence intervals)
☐	☒	For null hypothesis testing, the test statistic (e.g. F, t, r) with confidence intervals, effect sizes, degrees of freedom and P value noted<br>*Give P values as exact values whenever suitable.*
☒	☐	For Bayesian analysis, information on the choice of priors and Markov chain Monte Carlo settings
☐	☒	For hierarchical and complex designs, identification of the appropriate level for tests and full reporting of outcomes
☐	☒	Estimates of effect sizes (e.g. Cohen's d, Pearson's r), indicating how they were calculated

Our web collection on statistics for biologists contains articles on many of the points above.

Software and code

Policy information about availability of computer code

Data collection	Data from the videos was collected using Windows Media Player 12 (2013). RGB values of coloured almonds were measured using Adobe Photoshop Elements (2020).
Data analysis	Data analysis was conducted with the software R.3.6.1 using lme4 (version 1.1.21), asnipe (version 1.1.11) and NBDA (version 0.7.10) packages.

For manuscripts utilizing custom algorithms or software that are central to the research but not yet described in published literature, software must be made available to editors and reviewers. We strongly encourage code deposition in a community repository (e.g. GitHub). See the Nature Research guidelines for submitting code & software for further information.

Data

Policy information about availability of data

All manuscripts must include a data availability statement. This statement should provide the following information, where applicable:

- Accession codes, unique identifiers, or web links for publicly available datasets
- A list of figures that have associated raw data
- A description of any restrictions on data availability

All data is available in Dryad: https://doi.org/10.5061/dryad.9s4mw6mcv

Field-specific reporting

Please select the one below that is the best fit for your research. If you are not sure, read the appropriate sections before making your selection.

☐ Life sciences ☐ Behavioural & social sciences ☒ Ecological, evolutionary & environmental sciences

For a reference copy of the document with all sections, see nature.com/documents/nr-reporting-summary-flat.pdf

Ecological, evolutionary & environmental sciences study design

All studies must disclose on these points even when the disclosure is negative.

Study description

We investigated how great tits (Parus major) and blue tits (Cyanistes caeruleus) learn about novel palatable and unpalatable food in the wild. Birds were fitted with RFID tags and antennas at feeding stations enabled us to record each individual's visits. We first constructed a social network of the bird population based on their associations at the feeders (n = 191). To investigate avoidance learning, we presented birds with coloured palatable and unpalatable almonds using a paired-feeder design distributed across the study area. We replicated this with three colour pairs (unpalatable/palatable): red/green (n = 86), blue/purple (n = 90), and yellow/orange (n = 168). After blue/purple experiment, we also tested reversal learning by switching both colours to be palatable (n = 118). We investigated whether birds used social information during avoidance learning by calculating the expected number of observations from the social network and analysing birds' choices with generalized mixed models. Social information use during reversal learning was investigated using network-based diffusion analysis (NBDA).

Research sample

Research data includes records of foraging choices of wild great tits (Parus major) and blue tits (Cyanistes caeruleus) at an established field site in Madingley Wood, Cambridgeshire. Great tits and blue tits were used as study species because they are generalist predators and social foragers, and therefore ecologically relevant to our research question. The study site was chosen because of an ongoing research project of blue tit and great tit population in the area. This includes fitting birds with RFID tags that enabled us to record birds that were visiting the feeders. The sample represents foraging choices of adult and juvenile great tits and blue tits. During the study, 112 great tits (23 adults and 89 juveniles) and 79 blue tits (24 adults and 55 juveniles) visited the feeders, but the sample size for each learning experiment (different colour pair) varied (see above) as birds were foraging freely. Birds were classified to adults (> 1 year old) and juveniles (born that year), but we could dot determine their sex because blue tits and juvenile great tits cannot be sexed confidently based on plumage.

Sampling strategy

Birds were foraging freely in the field and we could not influence how many individuals visited the feeders and participated in each experiment. In each experiment, the sample size was sufficient (minimum 86 birds and 4410 records) to estimate the effect of asocial and social learning, and compare foraging choices of adults and juveniles.

Data collection

Visits to the feeders were monitored using RFID antennas and data loggers (Francis Scientific Instruments, Ltd) that scanned birds' unique RFID tag codes when they landed on a perch attached to the feeder. We also recorded videos from the feeding stations (using Go Pro Hero Action Camera and Canon Legria HF R66 Camcorder) and these were analysed by Liisa Hämäläinen.

Timing and spatial scale

Spatial scale: Experiments were conducted at Madingley Wood, Cambridgeshire, UK (0∘3.2´E, 52∘12.9´N). Birds were provided almond flakes in paired feeders at three locations that were approximately 170m from each other.

Timing: We collected social network data at the feeders throughout the summer 2018 (5th June - 17th September). During this time, we conducted four learning experiments:
- avoidance learning red/green: 5th July - 17 July
- avoidance learning blue/purple: 21st July - 30th July
- reversal learning blue/purple: 3rd August - 13th August
- avoidance learning yellow/orange: 25th August - 2nd September

Avoidance learning experiments were finished when > 90% of the visits were to the palatable feeder which indicated that birds had learned to discriminate the colours. The reversal learning experiment was finished when birds were visiting both feeders equally often, indicating that they had reversed their avoidance.

Data exclusions

No data was excluded from the analyses.

Reproducibility

We replicated avoidance learning experiment with three colour pairs and found consistent results of social information use. The experiment is reproducible following the description of methods in the manuscript. The data and the R codes for the analyses are publicly available.

Randomization

Randomization was not relevant to our study as we did not have different treatment groups.

Blinding

Data was collected with automated data loggers, so blinding was not relevant.

Did the study involve field work? ☒ Yes ☐ No

Field work, collection and transport

Field conditions

The study was conducted at an established 15.4 hectare field site in Madingley Wood during summer 2018 (5th June - 17th

Field conditions	September). Climatic conditions varied from day to day (average temperature 16C, average monthly rainfall 24mm), but this did not influence data collection.
Location	Madingley Wood, Cambridgeshire, UK (0∘3.2´E, 52∘12.9´N)
Access & import/export	Madingley Wood is an established field site and access to the site was granted by the University of Cambridge Estate Management. We did not import any samples.
Disturbance	During mist netting birds were handled and processed quickly by experienced ringers to minimize disturbance.

Reporting for specific materials, systems and methods

We require information from authors about some types of materials, experimental systems and methods used in many studies. Here, indicate whether each material, system or method listed is relevant to your study. If you are not sure if a list item applies to your research, read the appropriate section before selecting a response.

Materials & experimental systems

n/a	Involved in the study
☒	☐ Antibodies
☒	☐ Eukaryotic cell lines
☒	☐ Palaeontology and archaeology
☐	☒ Animals and other organisms
☒	☐ Human research participants
☒	☐ Clinical data
☒	☐ Dual use research of concern

Methods

n/a	Involved in the study
☒	☐ ChIP-seq
☒	☐ Flow cytometry
☒	☐ MRI-based neuroimaging

Animals and other organisms

Policy information about studies involving animals; ARRIVE guidelines recommended for reporting animal research

Laboratory animals	The study did not involve laboratory animals.
Wild animals	Great tits (n = 112: 23 adults and 89 juveniles) and blue tits (n = 79: 24 adults and 55 juveniles) were caught from feeding stations using mist nets, or caught from nest boxes when chicks were approximately 10 days old. We could not determine their sex (blue tits and juvenile great tits cannot be sexed confidently based on plumage). All birds were fitted with British Trust of Ornithology (BTO) ID rings and RFID tags before released at the capture site, or returned to the nest box.
Field-collected samples	The study did not involve samples collected from the field.
Ethics oversight	The study protocol was approved by the Animal Users Committee at the Department of Zoology, University of Cambridge. Authorisation for RFID tagging was granted by British Trust of Ornithology (BTO Special Methods permit to Hannah Rowland).

Note that full information on the approval of the study protocol must also be provided in the manuscript.
